# Draft Genome Sequence of Bradyrhizobium elkanii BR 2003, an Efficient Rhizobium Strain for *Cajanus, Canavalia*, *Crotalaria*, and *Indigofera*

**DOI:** 10.1128/MRA.01565-19

**Published:** 2020-03-12

**Authors:** Jerri Édson Zilli, Jean Luiz Simoes-Araujo, Luc Felicianus Marie Rouws, Luis Henrique de Barros Soares

**Affiliations:** a Embrapa Agrobiologia, Seropédica, Rio de Janeiro, Brazil; Loyola University Chicago

## Abstract

We report here the annotated draft genome sequence of the rhizobium strain BR 2003. This strain is able to establish symbiosis and to fix nitrogen with a broad range of leguminous species. The estimation of the average nucleotide identity confirmed the strain as a member of Bradyrhizobium elkanii.

## ANNOUNCEMENT

The genus *Bradyrhizobium* (alphaproteobacteria) comprises some efficient nitrogen-fixing bacteria that are able to nodulate leguminous species. Strains of this genus are frequently present in nodules of tropical and subtropical leguminous species, and strains that are well adapted to diverse edaphoclimatic conditions can be found in different biomes (1, 2).

For instance, strain BR 2003 was isolated in 1982 from nodules of Crotalaria juncea grown in Brazilian Cerrado soil (3). Field experiments have indicated that this strain is efficient in symbiotic nitrogen fixation with green manure species C. juncea, Crotalaria spectabilis, Cajanus cajan, Canavalia ensiformis, and Indigofera hirsuta (4). Here, we present the draft genome sequence of strain BR 2003, which is used in commercial inoculants for green manure in Brazil. The phylogeny constructed using 16S rRNA gene sequences showed that this strain is a member of the Bradyrhizobium elkanii clade, with high similarity to different related type strains.

Strain BR 2003 was originally isolated and purified on yeast extract-mannitol (YM) agar plates and was kept lyophilized for long-term storage after five passages on culture medium for purification. A single colony from a fresh plate was inoculated in liquid YM medium and grown for 4 days at 28°C with agitation (5). Genomic DNA was isolated from the stationary-phase bacterial culture using the Wizard DNA clean-up kit (Promega). A whole-genome paired-end library was constructed using the TruSeq PCR-free kit, and samples were run on a HiSeq 2500 sequencer (Illumina) at Macrogen (South Korea).

The Illumina sequencer generated a total of 18,045,344 paired-end reads (101 bp long), with 99.51% of the reads having a Phred quality score higher than 20. The reads were trimmed using the FASTX-Toolkit (http://hannonlab.cshl.edu/fastx_toolkit), and only bases with a quality score above 20 were used for *de novo* genome assembly using ABySS software version 2 (6). The kmer length parameter was tested from 70 to 98, and a genome with 124 contigs and 8,946,006 base pairs was the best result from the ABySS assembly, using a kmer value of 97. GFinisher software version 1.2 (7) was used to refine the BR 2003 genome ABySS assembly. GFinisher generates fuzzy GC content skew graphs for all contigs and compares them to a draft genome assembly using BLAST to find sequences that overlap gaps. The reassembly and contig/scaffold organization were carried out using Bradyrhizobium japonicum strain SEMIA 5079 (GenBank accession number NZ_CP007569.1) as a reference genome. Annotation and identification of metabolic pathways for the draft genome were performed using the RAST version 2.0 server (8). The genome sequence available at NCBI was annotated using the Prokaryotic Genome Annotation Pipeline (PGAP) version 3.1 (9). Default parameters were used for all software unless otherwise specified. The reassembly GFinisher summed 8.20 megabases, distributed in 52 contigs, with a contig *N*_50_ value of 298.6 kb. The size of the longest contig was 506.2 kb. The coverage reported by the assembler was 150×, and the genome GC content is 64.4%, which is within the range for the *Bradyrhizobium* genus. The RAST annotation identified 8,093 coding DNA sequences, distributed across 371 subsystems, and 48 RNAs. The largest number of genes was observed in the subsystem of amino acids and derivatives (629 genes), followed by carbohydrates; 51 genes were involved in nitrogen metabolism, such as nitrogen fixation (20 genes), nitrate and nitrite ammonification (11 genes), and ammonia assimilation (14 genes). However, no denitrification-related genes were found.

A high-resolution species tree generated by Automated Multi-Locus Species Tree software (10), using 83 marker genes, showed that BR 2003 was most closely related to Bradyrhizobium elkanii type strain USDA76 (GenBank accession number NZ_ARAG00000000.1). In fact, the estimated average nucleotide identity between BR 2003 and USDA76 was 96.12%, confirming that the strains belong to the same species. However, when compared with that of USDA76, the BR 2003 genome has 106 exclusive genes, including 12 related to the type VI secretion system (T6SS). The T6SS allows bacteria to translocate effector proteins to other bacteria or to eukaryotic cells and can act as a contact-dependent interbacterial “weapon” to keep competing strains from co-occupying sites in the host (11). Interestingly, in Azorhizobium caulinodans (strain ORS571), the T6SS seems to participate specifically in symbiosis by increasing the symbiotic competitiveness (12, 13). Other membrane transporter genes exclusive to BR 2003, including six genes from the ABC transporter family and five genes related to resistance to antibiotics and toxic compounds, were also detected; this suggests that BR 2003 has distinct capabilities with respect to the transport of specific molecules.

### Data availability.

The assembled genome sequence was deposited in GenBank under BioProject accession number PRJNA526863 (BioSample accession number SAMN11116018). The raw data accession number is SRX7288786. The version described in this paper is the first version under accession number SMYJ00000000, and the genome sequence available at NCBI was annotated using PGAP version 3.1 (9).
